# Impact of the COVID-19 pandemic on radiography practice: findings from a UK radiography workforce survey

**DOI:** 10.1259/bjro.20200023

**Published:** 2020-09-02

**Authors:** Theophilus N Akudjedu, Olanrewaju Lawal, Meera Sharma, Jason Elliott, Sharon Stewart, Terri Gilleece, Sonyia McFadden, James M Franklin

**Affiliations:** 1 Institute of Medical Imaging & Visualisation, Department of Medical Science & Public Health, Faculty of Health & Social Sciences, Bournemouth University, Bournemouth, UK; 2 Department of Allied Health Professions, Midwifery and Social Work, School of Health and Social Work, University of Hertfordshire, Hertfordshire, UK; 3 School of Nursing, Midwifery and Health, Coventry University, Coventry, UK; 4 School of Healthcare Sciences, Cardiff University, Cardiff, UK; 5 Department of Podiatry and Radiography, School of Health and Life Sciences, Glasgow Caledonian University, Glasgow, UK; 6 Institute of Nursing and Health Research, Ulster University, Ulster, UK

## Abstract

**Objectives:**

Radiographers are key patient-facing healthcare professionals involved in many aspects of patient care. The working patterns and professional practice of the radiography workforce (RW) has been altered during the COVID-19 pandemic. This survey aimed to assess the impact of the pandemic on radiography practice in the United Kingdom (UK).

**Methods:**

An online cross-sectional survey of the UK RW was performed (March 25th to April 26th, 2020). The survey sought information regarding 1. Demographics 2. Impact of the pandemic on professional practice 3. Infection prevention/control and 4. COVID-19 related stress. Data collected was analysed using the Statistical Package for Social Sciences (v.26).

**Results:**

A total of 522 responses were received, comprising *n* = 412 (78.9%) diagnostic and *n* = 110 (21.1%) therapeutic RW categories from across the UK. 12.5% (65/522) of the respondents were redeployed. Redeployment did not appear to contribute (*p* = 0.31) to work-related stress. However, fear of contracting the infection and perceived inadequate personal protective equipment (PPE) were identified as key contributors to stress during the study period. Compared to the therapeutic RW, a significantly higher proportion of the diagnostic RW identified fear of being infected as a major stressor (166/412 (40.3%) *vs* 30/110 (27.3%), *p* = 0.01).

**Conclusion:**

This survey has demonstrated changes to clinical practice, in particular to working patterns, service delivery and infection prevention and control were key contributors to workplace-related stress during the pandemic.

**Advances in knowledge:**

Timely and adequate staff training and availability of PPE as well as psychosocial support during future pandemics would enhance quality patient and staff safety.

## Introduction

In December 2019, cases of pneumonia with an unidentified origin in a cluster of patients emerging from the Chinese City of Wuhan were reported to the World Health Organisation (WHO).^1,2^ A few days after these reports, the novel severe acute respiratory syndrome coronavirus 2 [SARS-CoV-2]) was confirmed as the pathogenic cause of these cases, and the outbreak was subsequently named coronavirus disease (COVID-19).^2^ The first confirmed cases outside mainland China were reported to the WHO from Japan, South Korea and Thailand on January 20th, 2020.^3^ The disease was subsequently reported in various continents, including Europe, with the United Kingdom (UK) reporting two cases on January 29th, 2020. The disease has spread rapidly across the globe and has led to widespread implementation of lockdown measures in most countries and restrictions on both local and international travel. The WHO declared the outbreak as a health emergency on January 30th, 2020^3^ because of the rapidly increasing number of cases and deaths associated with the virus globally. There were 21,162,956 cases and 764,741 deaths reported worldwide, with 315,621 confirmed cases and 46,791 related deaths in the UK as at August 15th, 2020.^4^

Imaging, in particular chest radiographs (CXR) and computed tomography (CT), have emerged as key components of patient investigation and management pathways.^5–7^ The clinical radiography workforce (RW), including assistant practitioners (APs), diagnostic and therapeutic radiographers are crucial patient-facing staff responsible for most diagnostic image acquisition, and delivery of daily radiotherapy treatments. In the pandemic, they are often involved in acquiring CXRs or CTs as part of the care of patients with known or suspected COVID-19. However, other diagnostic and therapeutic work, including imaging patients with medical emergencies and delivering components of cancer imaging and treatment has continued during the peak of the pandemic.

The National Cancer Research Institute (NCRI) reported major changes in relation to radiotherapy treatment delivery due to the acute phase of the pandemic in the UK.^8^ This included an increase in the use of radiotherapy as first definitive treatment for cancer in response to reduction in surgical capacity during the pandemic. Of note, there has been rapid publication of national and international guidance on changing radiotherapy schedules,^9–12^ with differing levels of evidence to support the recommendations.^12,13^ Consequently, the case mix and protocols have also been altered in radiotherapy departments, with, for example, the widespread implementation of ‘Fast Forward’ protocols for patients with breast cancer.^12–15^

In an attempt to respond to the pandemic, the National Health Service (NHS) workforce has come under extreme pressure.^16^ The NHS pandemic response involved proactively postponing non-urgent work and screening programmes, redesign and relocation of services within hospitals and across regions, re-purposing of physical resources and redeployment (*i.e.,* using a different imaging modality and/or re-assignment) of clinical staff to cover anticipated acute care demands.^17,18^ Furthermore, a temporary register has been developed by the Health and Care Professions Council (HCPC) for automatic inclusion of all former registrants who have de-registered in the past three years and others, including final year radiography students on UK approved programmes who have completed clinical placements to join the workforce.^19^ This temporary workforce is expected to operate within the limits of their skills, knowledge, and experience.^20^

With growing global concerns about the pandemic and possibility of additional waves of infection, radiology departments have adopted several streamlined approaches towards practice to limit infection risk while optimising workflows, volumes and access.^7,21–23^ Of note, current recommendations emphasise the importance of appropriate use of personal protective equipment (PPE) and the implementation of strict infection prevention and control (IPC) protocols for the management of this pandemic.^7,21–25^ It is also essential for departments to prioritise the health and well-being of the workforce during the pandemic.^26–29^ Previous studies that investigated workplace-related stressors among radiographers cited staff shortages, heavy workload and volume of patients as some of the key sources of occupational stress.^30,31^ The current pandemic is likely to present additional workplace-related stressors for all patient-facing healthcare workers, including the RW, related to the risk of contracting the infection and changes in work patterns. The aim of this research is to assess the perceptions of the RW on the impact of the COVID-19 pandemic on practice in the UK.

## Methods

### Survey design and distribution

A cross-sectional survey of UK radiographers, APs and those on the temporary register was considered the most appropriate method to access the information required for this study. The research team representatives of the UK nations (England, Wales, Scotland and Northern Ireland) outlined the questions (Appendix 1) required for the survey. These included structured questions with few options for comments relating to 1. Demographics 2. Impact of the pandemic on professional practice 3. Infection prevention and control and 4. COVID-19 related stress. This survey is a part of the international study [COVID-19 Response in Radiology (CORIRA)] aimed at assessing the global impact of the COVID-19 pandemic on radiology workforce and practice. This arm of the study aimed to assess the perceptions of the RW on the pandemic in the UK; therefore, those on the temporary register and APs were eligible so far as they are engaged in current practice or volunteering during the pandemic. The academic RW is estimated at less than 2% of the overall therapeutic and diagnostic RW,^32^ and it was hypothesised that a part of this cohort may be redeployed as part of the pandemic response. Furthermore, some academic radiographers are normally involved in clinical service provision through joint clinical/academic contracts and thus, they were also included.

The survey was conducted online using Google forms (https://docs.google.com/forms/). Prior to distribution, the online survey was piloted by three members of the research team, with amendments made to ensure the questions were explicit and clear. The link to the online survey was shared amongst radiology health board and NHS Trust leads across the UK via email and was advertised on social media platforms to maximise the response rate. In addition, a network of colleagues’ personal contacts were also employed to promote the survey. The response time frame for the survey was four weeks (March 25th to April 26th, 2020) with weekly reminders on social media platforms. Due to the nature of the questionnaire which specifically asked about stress/anxiety relating to the pandemic and other concerns, a link was provided on completion of the survey to a support page developed by a certified clinical psychologist, which encouraged participants to engage with a self-help survival guide.^33^ Ethical approval was obtained from the Bournemouth University Research Ethics Committee (Ethics ID: 31818), and all the respondents provided electronic informed consent for participation in the study.

### Statistical analyses

The survey data was downloaded from Google forms into the Statistical Package for Social Sciences (SPSS) version 26.0 for Windows (SPSS Inc., IBM, New York, USA) for analyses.

Data obtained from the HCPC in accordance to the Freedom of Information Act 2000^34^ reported a total of 27,041 diagnostic and 4,616 therapeutic radiographers in May, 2018. Furthermore, a report from NHS England showed a steady growth rate for diagnostic radiographers, ranging between 3 and 4% per year over the period with similar projections to 2027.^35^ In an extrapolation analysis that assumed a 4% annual growth rate for the RW across the UK, it is suggested that there are approximately 34,241 radiographers [diagnostic (*n* = 29,248) and therapeutic (*n* = 4,993)] currently registered in the UK. Snaith and colleagues^36^ previously reported the contributions of APs in easing staff shortages and facilitation of efficient service delivery. The Society and College of Radiographers (SCoR) states there are 189 voluntarily accredited APs across diagnostic and therapeutic radiography practice on their register in June, 2020 (personal communication, M Landau). Using the Qualtrics^®^ online sample size calculator (https://www.qualtrics.com/blog/calculating-sample-size/), the estimated required sample size for the study was a minimum of 380 valid responses.

*χ*^*2*^ tests were used to investigate the relationship between the demographic variables across the RW categories (diagnostic *vs* therapeutic). Again, it was used to assess the impact of redeployment on perceived stress levels among the workforce. A two-tailed α level of 0.05 was used for testing statistical significance.

Finally, a directed content analysis was deemed appropriate for the free text comments, as the respondents’ views were mostly placed within suitable predetermined categories.^37^

## Results

### Response rate and demographics

Detailed data on demography, workplace setting, professional status and geographical location of respondents are presented in Table 1. Within the four-week study period, this survey recorded 522 valid responses. The respondents comprise of the diagnostic (412/522; 78.9%) and therapeutic (110/522; 21.1%) RW categories from across all the UK nations with the highest response (317/522; 60.7%) from England. Of the respondents, 403 (of 522; 77.2%) were female and only 18 (of 522; 3.4%) were aged 60 years and above. Radiographers on the temporary register accounted for 14 (of 522; 2.7%) of the valid responses. A chi-square test of independence showed a significant relationship between the radiography categories and sex (*p* = 0.01), age group (*p* = 0.04) and workplace setting (*p* = 0.0004).

**Table 1.** Demography, workplace setting and geographical location of respondents

Variables	Groups	Category of Radiography Workforce				Total	
		Diagnostic		Therapeutic			
		*Head count (n)*	*%*	*Head count (n)*	*%*	*Head count (n)*	*%*
Sex	Female	307	74.5	96	87.3	403	77.2
	Male	103	25.0	13	11.8	116	22.2
	Prefer not to say	2	0.5	1	0.9	3	0.6
Age group	18–29 years old	116	28.2	28	25.5	144	27.6
	30–39 years old	126	30.6	21	19.1	147	28.2
	40–49 years old	107	26.0	33	30.0	140	26.8
	50–59 years old	50	12.1	23	20.9	73	14.0
	60 years and above	13	3.2	5	4.5	18	3.4
	Prefer not to say	0	0	0	0	0	0
Workplace Setting	Public: Urban Setting	205	49.8	48	43.6	253	48.5
	Public: Community Setting	87	21.1	38	34.5	125	23.9
	Public: Rural/District Setting	73	17.7	11	10.0	84	16.1
	Public: University/Academic Setting^*a*^	40	9.7	6	5.5	46	8.8
	Private Facility	7	1.7	5	4.5	12	2.3
	Others^*b*^	0	0	2	1.8	2	0.4
Geographical Location	England	243	59.0	74	67.3	317	60.7
	Wales	48	11.7	9	8.2	57	10.9
	Scotland	83	20.1	20	18.2	103	19.7
	Northern Ireland	38	9.2	7	6.4	45	8.6
Professional Status	Registered Radiographer	394	95.6	109	99.1	503	96.4
	Assistant Practitioner	4	1.0	1	0.9	5	1.0
	Temporary Registered (student)	13	3.2	0	0	13	2.5
	Temporary Registered (retired)	1	0.2	0	0	1	0.2
**Total Head count (n**)		**412**		**110**		**522**	

a Three (0.6%) respondents indicated they hold a joint clinical/academic role with their primary place of work in Public: University/Academic Setting.

b charities, device manufacturing companies etc.

### Perspectives of the radiography workforce on the impact of COVID-19 on practice

Table 2 shows that a higher proportion of respondents (447/522; 85.7%) strongly agree that radiographers are a part of the major frontline healthcare management team in response to the pandemic. The workload of 196 (of 522; 37.5%) respondents was reported as increasing during the survey period. A total of 65 (of 522; 12.5%) respondents [diagnostic: (62/412; 15.0%) and therapy: (3/110; 2.7%)] have been redeployed to use other imaging/therapy modalities during the study period (Table 2). The free-text comments provided by respondents were reviewed for detailed description of changes in workload and the general working environment. Sample responses highlighted the themes of staff redeployment, workload redistribution/shift mostly at the departmental level and perceived general workload increase across the RW (mainly due to the strict adherence to IPC protocols, see comments relating to IPC).

**Table 2.** Participants’ response to survey

Statement/ Question	Category of Radiography Workforce	Response, n (%)						
***1. Perspectives of radiographers on the impact of COVID-19 on practice***								
Radiographers are a part of the major frontline healthcare management team in response to COVID-19.		Strongly agree	Agree	Neutral		Disagree		Strongly disagree
	DR	371 (90.0%)	1 (0.2%)	2 (0.5%)		2 (0.5%)		36 (8.7%)
	TR	76 (69.1%)	0	4 (3.6%)		3 (2.7%)		27 (24.5%)
	Total	447 (85.6%)	1 (0.2%)	6 (1.1%)		5 (1.0%)		63 (12.1%)
Which of the following best describes your workload pattern after the COVID-19 outbreak in the UK?		Increasing pattern	Decreasing pattern	Not changed		Irregular pattern		Other (free text description)
	DR	159 (38.6%)	108 (26.2%)	21 (5.1%)		122 (29.6%)		2 (0.5%)
	TR	37 (33.6%)	31 (28.2%)	20 (18.2%)		22 (20.0%)		0
	Total	196 (37.5%)	139 (26.6%)	41 (7.9%)		144 (27.6%)		2 (0.4%)
Have you had to use other imaging modalities apart from the ones you use for your daily work after the COVID-19 outbreak?		Yes			No			
	DR	62 (15.0%)			350 (85.0%)			
	TR	3 (2.7%)			107 (97.3%)			
	Total	65 (12.5%)			457 (87.5%)			
***2. Radiographers understanding of COVID-19 transmission, infection control and availability of PPE***								
		Strongly agree	Agree	Neutral		Disagree		Strongly disagree
I have a great understanding of how the COVID-19 virus is transmitted.	DR	132 (32.0%)	211 (51.2%)	48 (11.7%)		17 (4.1%)		4 (1.0%)
	TR	39 (35.5%)	54 (49.1%)	9 (8.2%)		7 (6.4%)		1 (0.9%)
	Total	171 (32.8%)	265 (50.8%)	57 (10.9%)		24 (4.6%)		5 (1.0%)
My understanding of the principles of infection prevention and control as a radiographer is adequate to deal with the COVID-19 outbreak.	DR	42 (10.2%)	215 (52.2%)	60 (14.6%)		32 (7.8%)		63 (15.3%)
	TR	18 (16.4%)	51 (46.4%)	17 (15.5%)		8 (7.3%)		16 (14.5%)
	Total	60 (11.5%)	266 (51.0%)	77 (14.8%)		40 (7.7%)		79 (15.1%)
My facility has made available adequate personal protective equipment (PPE) for work during the COVID-19 outbreak.	DR	52 (12.6%)	158 (38.3%)	73 (17.7%)		86 (20.9%)		43 (10.4%)
	TR	17 (15.5%)	36 (32.7%)	17 (15.5%)		25 (22.7%)		15 (13.6%)
	Total	69 (13.2%)	194 (37.2%)	90 (17.2%)		111 (21.3%)		58 (11.1%)
		Yes			No			
Have you had any training specifically to prepare you for handling patients during the COVID-19 outbreak?	DR	204 (49.5%)			208 (50.5%)			
	TR	48 (43.6%)			62 (56.4%)			
	Total	252 (48.3%)			270 (51.7%)			
***3. Profile of COVID-19 related stress, it’s impact and available support systems***								
Do you feel stressed about work lately due to the COVID-19 outbreak?		Yes, always		Sometimes				No
	DR	260 (63.1%)		116 (28.2%)				36 (8.7%)
	TR	70 (63.6%)		34 (30.9%)				6 (5.5%)
	Total	330 (63.2%)		150 (28.7%)				42 (8.0%)
I feel I may be in need of professional help to deal with stress during the COVID-19 outbreak.		Strongly agree	Agree	Neutral			Disagree	Strongly disagree
	DR	15 (3.6%)	65 (15.8%)	147 (35.7%)			140 (34.0%)	45 (10.9%)
	TR	5 (4.5%)	17 (15.5%)	42 (38.2%)			31 (28.2%)	15 (13.6%)
	Total	20 (3.8%)	82 (15.7%)	189 (36.2%)			171 (32.8%)	60 (11.5%)
My family/partner/friends are being significantly affected by this recent work-related stress.	DR	43 (10.4%)	170 (41.3%)	90 (21.8%)			79 (19.2%)	30 (7.3%)
	TR	17 (15.5%)	39 (35.5%)	24 (21.8%)			17 (15.5%)	13 (11.8%)
	Total	60 (11.5%)	209 (40.0%)	114 (21.8%)			96 (18.4%)	43 (8.2%)
There are adequate social and psychological support structures at work for dealing with stress.	DR	22 (5.3%)	125 (30.3%)	133 (32.3%)			106 (25.7%)	26 (6.3%)
	TR	13 (11.8%)	35 (31.8%)	28 (25.5%)			19 (17.3%)	15 (13.6%)
	Total	35 (6.7%)	160 (30.7%)	161 (30.8%)			125 (23.9%)	41 (7.9%)

The total valid survey response = 522, comprising of diagnostic radiography workforce (DR)(*n* = 412) and therapy radiography workforce (TR)(*n* = 110).

“*Increased workload pattern with COVID-19 cases however reduced work pattern for non-COVID-19 cases.*”

[Respondent ID: 211, diagnostic radiographer]

“*Reduced workload for routine outpatient work. Increased workload for general X-ray/CT inpatients. Radiographers being redeployed to assist in wards due to decreased workload in specialist modalities that mostly undertake 'routine' work.*”

[Respondent ID: 108, diagnostic radiographer]

“*Screening ceased and redeployed to general radiography to do COVID cases*.”

[Respondent ID: 178, Assistant Practitioner]

“*Loss of one treatment unit due to lack of workspace in console means increased workload on all other treatment units*.”

[Respondent ID: 309, therapeutic radiographer]

Within the survey period, most respondents reported increased pressure on procedural volumes of diagnostic [CXR (385/412), CT (264/412)] and therapeutic [Linear accelerator (LINAC) (64/110) services.

### Understanding of COVID-19 transmission, infection control and availability of PPE

Responses indicate that 436 (of 522; 83.6%) of the RW agreed [strongly agree: (171/522; 32.8%) or agree: (265/522; 50.8%)] that they have a good understanding of the mode of transmission of the virus (Table 2). Furthermore, 326 (of 522; 62.5%) Of the respondents, agree [strongly agree: (60/522; 11.5%) or agree: (266/522; 51%)] that their current level of understanding of the principles of IPC was adequate to deal with the outbreak (Table 2). Less than half (252/522; 48.3%) reported receiving specific training for safe handling of COVID-19 patients during the study period. Of the respondents, only half (263/522; 50.4%) agreed [strongly agree: (69/522; 13.2%) or agree (194/522; 37.2%)] that there were adequate PPE available for use at work during the study period. A common theme from the free text comments suggest adherence to increased infection prevention and control protocols which has consequently increased examination times per patient.

“*…there is a greater requirement for cleaning and the donning and doffing of PPE all adds to the workload, increasing the examination time per patient.*”

[Respondent ID: 412, diagnostic radiographer]

“*…. there is increased frequency of general cleaning and minimal patient waiting times within our department..*.”

[Respondent ID: 193, diagnostic radiographer]

### Profile of COVID-19 related stress, it’s impact and available support systems

**Figure 1.**
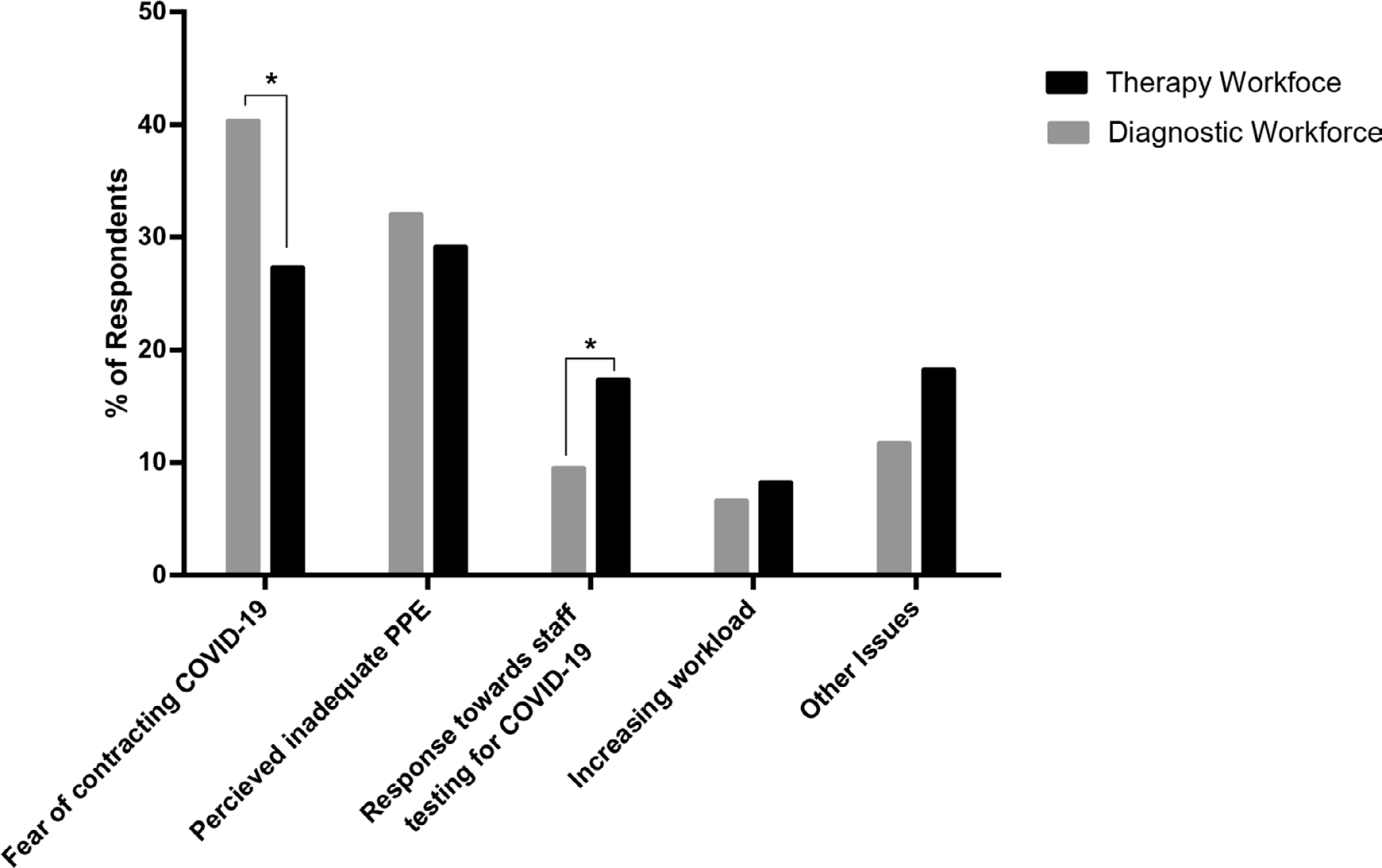
Distribution of some of the major stressors at work during the study period. Fear of contracting the infection and perceived inadequacy of PPE were considered the major stressors by the RW during the study period. Compared to the therapeutic RW, a significantly higher proportion of the diagnostic RW identified fear of being infected as a major stressor (166/412 (40.3%) *vs* 30/110 (27.3%), *p* = 0.01). However, compared to the diagnostic RW, a significantly higher proportion of the therapeutic RW were concerned about staff testing for COVID-19 (19/110 (17.3%) *vs* 39/412 (9.5%), *p* = 0.02). Other issues here, is inclusive of neutral and/or negative comments/responses in nature.

As shown in Table 2, 330 (of 522; 63.2%) respondents reported experiencing workplace-related stress after the outbreak. Overall, the perceived levels of stress did not differ significantly (*p* = 0.57) between the diagnostic and therapy RW categories. Furthermore, there was no significant difference between the RW categories across the proportion of respondents who perceived their work-related stress as low [0–4] (*p* = 0.72), intermediate [5-7](*p* = 0.41) or high [8-10] (*p* = 0.68) on the 0 (low stress) – 10 (extreme stress) Likert scale (Table 3). Figure 1 shows that fear of contracting the infection and perceived inadequacy of PPE were considered the major stressors by the RW during the study period. Compared to the therapeutic RW, a significantly higher proportion of the diagnostic RW identified fear of being infected as a major stressor (166/412 (40.3%) *vs* 30/110 (27.3%), *p* = 0.01; Figure 1). However, compared to the diagnostic RW, a significantly higher proportion of the therapeutic RW were concerned about staff testing for COVID-19 (19/110 (17.3%) *vs* 39/412 (9.5%), *p* = 0.02; Figure 1). In addition, a review of free-text comments suggested staff redeployment as a dominant theme for the increase in the current perceived work-related stress.

**Table 3.** Perceived stress among the radiography workforce during the study period

Level of stress^*a*^ (Likert Scale)	Stress Grading	Radiography Workforce Categories				Total	
		Diagnostic		Therapeutic			
		*Headcount (n)*	*%*	*Headcount (n)*	*%*	*Headcount (n)*	*%*
0	Low	5	1.2	2	1.8	7	1.3
1		5	1.2	1	0.9	6	1.1
2		39	9.5	14	12.7	53	10.2
3		16	3.9	2	1.8	18	3.4
4	Intermediate	11	2.7	4	3.6	15	2.9
5		18	4.4	2	1.8	20	3.8
6		20	4.9	8	7.3	28	5.4
7		56	13.6	10	9.1	66	12.6
8	High	92	22.3	32	29.1	124	23.8
9		105	25.5	25	22.7	130	24.9
10		45	10.9	10	9.1	55	10.5
Total		412	100	110	100	522	100

There was no significant difference between the radiography workforce categories across the proportion of respondents who perceived their work-related stress as low (*p* = 0.72), intermediate (*p* = 0.41) or high (*p* = 0.68).

a 0 = no stress; 10 = extreme stress.

“*My job has completely changed as a Mammographer; I am expected to return to general radiography after working in breast screening for ten years. I feel de-skilled in general, especially with advancements in digital equipment. This is a huge ask for mammographers to change their working environment at a time of increased pressure and stress!*”

*[Respondent ID: 176, diagnostic radiographer*]

Of the respondents, 160 (of 522; 30.7%) agreed that there are adequate social and support structures available at work for stress management during the study period (Table 2). However, only 171 (of 522; 32.8%) of the respondents disagreed that they would need professional help for the management of their recent workplace-related stress and 209 (of 522; 40.0%) of the respondents agree that the current workplace-related stress is having a significant impact on their family and friends (Table 2).

Considering that redeployment (*i.e.,* use of a different modality or re-assignment) emerged as a contributing factor to the perceived workplace-related stress during the pandemic, we investigated the relationship between redeployment and perceived levels of stress and its impact on family and friends. We observed that the perceived stress level of respondents did not differ across the workforce by redeployment during the study period, (*p* = 0.31) and did not significantly impact family/partners/friends (*p* = 0.35).

## Discussion

This national study is the first to comprehensively survey the RW on the perceived impact of the COVID-19 pandemic on their practice in the UK. Medical imaging emerged as a key clinical decision tool in the diagnosis, triaging for appropriate treatment pathways^5,6^ and repeat evaluation of the severely ill patients.^38^ Almost all the RW strongly agreed that radiographers are essential frontline staff in the management of COVID-19 patients.

Patterns of work changed during the pandemic. The diagnostic RW reported an increase in procedural volumes of CXR (385/412; 93.4%) and CT (264/412; 64.1%) during the study period, although overall volumes of diagnostic imaging have been lower, given the impact of the pandemic on elective care. Data from North America and Europe indicated there was a general decline in imaging procedural volumes, although, CXR and CT tended to be among the least affected.^39^ Our findings further indicate that increased PPE use and equipment decontamination have contributed to increased examination times and workload per patient.

Within the survey period, 58.2% (64/110) of the therapy RW perceived an increased pressure on procedural volume of the LINAC and changes in radiotherapy delivery. Findings from the Phase 3, randomised control trial^12^ for hypofractionated breast radiotherapy indicate the safe use of 26 Gy in 5 fractions over 1 week relative to 40 Gy in 15 fractions over 3 weeks for local tumour control. These timely findings informed recommendations for the radiotherapy dose-fractionation schedules during the pandemic. The perceived relative increase in procedure volumes at some radiotherapy departments during the peak of the pandemic, potentially reflect deviations from standard practice^9–12,14^ for some cancer patients who would have normally been on multimodality treatment regimens including surgery or chemotherapy. However, this finding is contrary to another similar survey^40^ which reported increased spare LINAC capacity since the pandemic in the UK. This inconsistency may be due to the different durations of these surveys, considering the quickly evolving nature of the pandemic as well as management guidelines.

In line with guidance to minimise non-urgent work,^17,18^ routine imaging services for diagnostic screening including mammography, MRI, nuclear medicine and ultrasound were paused in many units. Furthermore, the increase in procedural volume of CXR and CT scanning lead to rota changes and consequently staff redeployment from other units such as those involved in routine screening. Of the respondents, 65 (of 522; 12.5%) reported being redeployed, mostly to CXR and CT units to create extra capacity in response to the surge in COVID-19 patients and/or as an alternative to furlough as their usual specialist units were closed. Additional diagnostic capacity was provided from the temporary register with the addition of 14 radiographers to the workforce during the study period. The recruitment flexibility provided by the HCPC is highly recommended in the response strategy for future health emergencies. Staff redeployment emerged from the qualitative content analyses as a contributing factor to the perceived stress of the workforce who have to adjust to new work protocols, environments and technology. However, further statistical analyses indicated that the perceived stress levels among the workforce did not differ significantly by redeployment during the study period.

Infection control and PPE were and remain major sources of concern nationally and internationally during the pandemic.^21–25^ In patient-facing environments, such as radiology departments, precautions to limit contamination between patients and/or other professionals in the management of suspected or confirmed COVID-19 patients, including strict adherence to a PPE protocol, is strongly advocated.^7,22,25^ A large proportion of the RW (436/522; 83.6%) reported to have good knowledge of disease transmission, including the potential of cross-infection within the radiology department. This is broadly consistent with other studies^41–43^ that similarly reported a higher understanding of infection prevention, control and compliance among healthcare workers. However, only 62.5% (326/522) of the workforce considered their level of understanding as adequate for handling patients with known or suspected infection during the pandemic. This discordance potentially implies that specific training relating to infection control for COVID-19 was required during the acute phase of the pandemic. Staff training is important to prevent hospital-related transmission of the disease and as a requirement for continuous professional development in readiness for future pandemics. In this study, only less than half (252/522; 48.3%) of the respondents received specific guidance and training for safe handling of patients with known or suspected COVID-19 at work during the study period. With respect to PPE, only half (263/522; 50.4%) of the respondents perceived access to PPE required for work as adequate, which is in line with other similar surveys^40,44^ conducted during the pandemic among a broad array of radiology staff, including imaging and oncology professionals in the UK. Perceived uncertainty across this professional body is a potential consequence of the rapidly evolving practice and guidelines with respect to patient handling and PPE during the survey period, and also due to different practices across the NHS during the critical phase of the pandemic. Our findings indicate that changes in practice and IPC protocols contributed to the perceived stress for most of the RW during the study period. Although strict adherence to these protocols (including increased PPE use and equipment decontamination) are fundamental for keeping both patients and the workforce safe.^7,21–25^

The pandemic has presented a working environment of flux and uncertainty with major changes to routine departmental protocols and this is likely to have contributed to the perceived general increase in work-related stress. Previous studies showed that epidemics can lead to the development of new or worsening psychiatric symptoms such as fear, anxiety, panic attacks and depression.^27^ In the UK context, a recent survey demonstrated greater levels of coronavirus specific stress and anxiety within the general population, although they appeared mostly resilient in the initial stages of the pandemic.^45^ However, most respondents (330/522; 63.2%) of the current study reported to have started experiencing workplace-related stress during the pandemic. This perceived stress did not differ significantly between diagnostic and therapy RW categories. However, about 60% (309/522) of the RW rated their perceived stress levels to be high during the study period. Similarly, medical professionals in China reported elevated levels of stress and anxiety during this outbreak^26,27^. Surprisingly, only 30.7% (160/522) of the workforce agreed that there are adequate psychosocial support structures at work for the management of stress. Thus, it is important that institutional structures are strengthened to mitigate the impact of pandemic related stressors on both physical and psychological wellbeing.^26–29,46^ Previous studies^26,28^ suggested timely training and regular evaluation of stress, depression, and anxiety among health workers involved in the care of patients/victims during pandemics. In response to the pandemic, the SCoR^47^ developed a rapid training programme with experiences from disaster response, crisis psychology and human performance under conditions of extreme stress for use by its members as part of health, safety and well-being management.

We acknowledge a number of strengths and limitations associated with this study. To the best of our knowledge, this is the first and largest survey that comprehensively assessed the impact of the pandemic on radiography practice in a highly representative national sample of the RW recruited over a relatively long period during the pandemic. Our response rate (the required minimum sample size of valid responses was achieved) is considered satisfactory, although, there are varied response rates for different types of surveys,^48^ no clear standards exist for acceptable response rates, specifically for online surveys.^49^ The gender distribution of the respondents is identical to the entire NHS workforce with a 77% female composition.^50^ Furthermore, the geographical distribution of the RW obtained from this study is comparable to the population distribution of the UK, with the least workforce in Northern Ireland. The sample is representative of the RW on demographic, professional status, workplace setting and geographical location and thus, our findings can be considered robust and broadly transferable. Limitations associated with the current study include use of a non-standardised stress rating (simple Likert) scale, lack of a baseline assessment and our inability to quantify the actual changes in procedural volumes of the various imaging modalities over the survey period. Future studies would benefit from the use of standardised stress assessment tools for easy comparison to other studies.

## Conclusions

This survey has highlighted the important patient-facing role of the RW during the COVID-19 pandemic. Changes to clinical practice, in particular to working patterns, service delivery and IPC protocols were key contributors to workplace-related stress during the pandemic. Radiology departments should, therefore, seek to mitigate the impact on their workforce, through consistent communication, ongoing education, and provision of clear IPC guidance and PPE as well as strengthen institutional structures for the management of workplace-related stress in readiness for future pandemics.

## References

[1] World Health Organization. Pneumonia of unknown cause: China. 2020 Available from: www.who.int/csr/don/05-january-2020-pneumonia-of-unkown-cause-china/en/ [2020 April 20].

[2] World Health Organization. Coronavirus disease (COVID-19) technical guidance: The Unity Studies: Early investigations. 2020 Available from: www.who.int/emergencies/diseases/novel-coronavirus-2019/technical-guidance/early-investigations [2020 April 20].

[3] British Foreign Policy Group. COVID-19 Timeline. 2020 Available from: https://bfpg.co.uk/2020/03/covid-19-timeline/ [cited 2020 April 20].

[4] Coronavirus Resource Centre. COVID-19 Dashboard by the Centre for Systems Science and Engineering at Johns Hopkins University. 2020 Available from: https://coronavirus.jhu.edu/map.html [2020 July 18].

[5] Nair A, Rodrigues JCL, Hare S, Edey A, Devaraj A, Jacob J, et al A British Society of thoracic imaging statement: considerations in designing local imaging diagnostic algorithms for the COVID-19 pandemic. Clin Radiol 2020; 75: 329–34. doi: 10.1016/j.crad.2020.03.008 32265036 PMC7128118

[6] Rubin GD, Ryerson CJ, Haramati LB, Sverzellati N, Kanne JP, Raoof S, et al The role of chest imaging in patient management during the COVID-19 pandemic: a multinational consensus statement from the Fleischner Society. Chest 2020;. 10.1016/j.chest.2020.04.003 PMC7138384 32275978

[7] Stogiannos N, Fotopoulos D, Woznitza N, Malamateniou C COVID-19 in the radiology department: what radiographers need to know. Radiography 2020; 26: 254–63. doi: 10.1016/j.radi.2020.05.012 32532596 PMC7269964

[8] National Cancer Research Institute. NCRI’s CTRad to lead COVID RT: a UK-wide initiative to study the impact of COVID-19 on radiotherapy services and patient outcomes. 2020 Available from: https://www.ncri.org.uk/news/covid19-radiotherapy-initiative/ [2020 July 8].

[9] Marijnen CAM, Peters FP, Rödel C, Bujko K, Haustermans K, Fokas E, et al International expert consensus statement regarding radiotherapy treatment options for rectal cancer during the COVID 19 pandemic. Radiother Oncol 2020; 148: 213–5. doi: 10.1016/j.radonc.2020.03.039 32342861 PMC7194592

[10] Thomson DJ, Palma D, Guckenberger M, Balermpas P, Beitler JJ, Blanchard P, et al Practice recommendations for Risk-Adapted head and neck cancer radiation therapy during the COVID-19 pandemic: an ASTRO-ESTRO consensus statement. Int J Radiat Oncol Biol Phys 2020; 107: 618–27. doi: 10.1016/j.ijrobp.2020.04.016 32302681 PMC7194855

[11] Troost EGC, Nestle U, Putora PM, Bussink J Practice recommendations for lung cancer radiotherapy during the COVID-19 pandemic: an ESTRO-ASTRO consensus statement. Radiother Oncol 2020; 147: 227–8. doi: 10.1016/j.radonc.2020.04.030 32342862 PMC7194725

[12] Murray Brunt A, Haviland JS, Wheatley DA, Sydenham MA, Alhasso A, et al.START Trialists' Group Hypofractionated breast radiotherapy for 1 week versus 3 weeks (FAST-Forward): 5-year efficacy and late normal tissue effects results from a multicentre, non-inferiority, randomised, phase 3 trial. Lancet 20202008; 395371: 16131098–26107. doi: 10.1016/S0140-6736(20)30932-6 PMC7262592 32580883

[13] , Bentzen SM, Agrawal RK, Aird EGA, Barrett JM, Barrett-Lee PJ, et al.START Trialists' Group The UK standardisation of breast radiotherapy (start) trial B of radiotherapy hypofractionation for treatment of early breast cancer: a randomised trial. Lancet 2008; 371: 1098–107. doi: 10.1016/S0140-6736(08)60348-7 18355913 PMC2277488

[14] Coles CE, Aristei C, Bliss J, Boersma L, Brunt AM, Chatterjee S, et al International guidelines on radiation therapy for breast cancer during the COVID-19 pandemic. Clin Oncol 2020; 32: 279–81. doi: 10.1016/j.clon.2020.03.006 PMC7270774 32241520

[15] Coles C Guidelines on radiation therapy for breast cancer during the COVID-19 pandemic. 2020 Available from: https://www.rcr.ac.uk/sites/default/files/breast-cancer-treatment-covid19.pdf [2020 July 16]. 10.1016/j.clon.2020.03.006 PMC7270774 32241520

[16] BBC News. BBC Coronavirus: Up to fifth of UK workers “could be off sick at same time”. 2020 Available from: https://www.bbc.co.uk/news/uk-51718917 [2020 April 20].

[17] Scottish Government. Health screening programmes paused. 2020 Available from: https://www.gov.scot/news/health-screening-programmes-paused/ [2020 April 25].

[18] Department of Health. Temporary pause of routine screening programmes. 2020 Available from: https://www.health-ni.gov.uk/news/temporary-pause-routine-screening-programmes [2020 April 25].

[19] Health and Care Professions Council. Temporary Register. 2020 Available from: https://www.hcpc-uk.org/covid-19/temporary-register/ [2020 June 25].

[20] Care Quality Commission. COVID-19: response from IR(ME)R inspectorates. 2020 Available from: https://rqia.org.uk/RQIA/media/RQIA/Guidance/Covid-19-IRMER-National-Response-Updated-v3.pdf.

[21] Mossa-Basha M, Medverd J, Linnau K, Lynch JB, Wener MH, Kicska G, et al Policies and guidelines for COVID-19 preparedness: experiences from the University of Washington. Radiology 2020;: 201326. doi: 10.1148/radiol.2020201326 32687455

[22] Zanardo M, Martini C, Monti CB, Cattaneo F, Ciaralli C, Cornacchione P, et al Management of patients with suspected or confirmed COVID-19, in the radiology department. Radiography 2020; 26: 264–8. doi: 10.1016/j.radi.2020.04.010 32340912 PMC7167552

[23] Tsou IYY, Liew CJY, Tan BP, Chou H, Wong SBS, Loke KSH, et al Planning and coordination of the radiological response to the coronavirus disease 2019 (COVID-19) pandemic: the Singapore experience. Clin Radiol 2020; 75: 415–22. doi: 10.1016/j.crad.2020.03.028 32291080 PMC7151533

[24] World Health Organisation. Infection Prevention and Control (IPC) for Novel Coronavirus (COVID-19). 2020 Available from: https://openwho.org/courses/COVID-19-IPC-EN [2020 April 20]. 10.1093/jacamr/dlaa027 PMC7184425 32363343

[25] Nakajima K, Kato H, Yamashiro T, Izumi T, Takeuchi I, Nakajima H, et al COVID-19 pneumonia: infection control protocol inside computed tomography suites. Jpn J Radiol 2020; 38: 391–3. doi: 10.1007/s11604-020-00948-y 32185669 PMC7089199

[26] Rana W, Mukhtar S, Mukhtar S Mental health of medical workers in Pakistan during the pandemic COVID-19 outbreak. Asian J Psychiatr 2020; 51: 102080. doi: 10.1016/j.ajp.2020.102080 32283512 PMC7139243

[27] Montemurro N The emotional impact of COVID-19: from medical staff to common people. Brain Behav Immun 2020; 87: 23–4. doi: 10.1016/j.bbi.2020.03.032 32240766 PMC7138159

[28] Xiang Y-T, Yang Y, Li W, Zhang L, Zhang Q, Cheung T, et al Timely mental health care for the 2019 novel coronavirus outbreak is urgently needed. Lancet Psychiatry 2020; 7: 228–9. doi: 10.1016/S2215-0366(20)30046-8 32032543 PMC7128153

[29] Li Z, Ge J, Yang M, Feng J, Qiao M, Jiang R, et al Vicarious traumatization in the general public, members, and non-members of medical teams Aiding in COVID-19 control. Brain Behav Immun 2020; 88: 916–9. doi: 10.1016/j.bbi.2020.03.007 32169498 PMC7102670

[30] Verrier W, Harvey J An investigation into work related stressors on diagnostic radiographers in a local district hospital. Radiography 2010; 16: 115–24. doi: 10.1016/j.radi.2009.09.005

[31] Rutter DR, Lovegrove MJ Occupational stress and its predictors in radiographers. Radiography 2008; 14: 138–43. doi: 10.1016/j.radi.2006.09.008

[32] Knapp KM, Wright C, Clarke H, McAnulla SJ, Nightingale JM The academic radiography workforce: age profile, succession planning and academic development. Radiography 2017; 23 Suppl 1: S48–52. doi: 10.1016/j.radi.2017.05.012 28780951

[33] Cooper J Corona-Anxiety your self-help survival guide. 2020 Available from: https://www.gcu.ac.uk/media/documents/Corona-Anxiety-GCU.pdf.

[34] legislation.gov.uk. Freedom of Information Act 2000. 2020 Available from: http://www.legislation.gov.uk/ukpga/2000/36/section/8 [2020 July 2].

[35] National Health Service. Facing the Facts, Shaping the Future: a draft health and care workforce strategy for England to 2027. 2020 Available from: https://www.hee.nhs.uk/sites/default/files/documents/Facing%20the%20Facts,%20Shaping%20the%20Future%20%E2%80%93%20a%20draft%20health%20and%20care%20workforce%20strategy%20for%20England%20to%202027.pdf.

[36] Snaith B, Harris MA, Palmer D A UK survey exploring the assistant practitioner role across diagnostic imaging: current practice, relationships and challenges to progression. Br J Radiol 2018; 91: 20180458. doi: 10.1259/bjr.20180458 30004807 PMC6475955

[37] Hsieh H-F, Shannon SE Three approaches to qualitative content analysis. Qual Health Res 2005; 15: 1277–88. doi: 10.1177/1049732305276687 16204405

[38] British Thoracic Society. COVID-19: Information for The Respiratory Community; Better Lung Health for All. 2020 Available from: https://brit-thoracic.org.uk/about-us/covid-19-information-for-the-respiratory-community/ [2020 May 15].

[39] AuntMinnie.com. Pandemic paralysis: COVID-19 has major impact on imaging. 2020 Available from: https://www.auntminnie.com/index.aspx?sec=nws&sub=rad&pag=dis&ItemID=128865 [2020 April 20].

[40] Radiotherapy A Impact of COVID-19 on UK Radiotherapy. 2020 Available from: https://ebf9be9c-890d-4dca-b67e-2c40c584e614.filesusr.com/ugd/b68571_5a27d1bd9d434ebb898facc3199de2e8.pdf.

[41] Desta M, Ayenew T, Sitotaw N, Tegegne N, Dires M, Getie M Knowledge, practice and associated factors of infection prevention among healthcare workers in Debre Markos referral Hospital, Northwest Ethiopia. BMC Health Serv Res 2018; 18: 465. doi: 10.1186/s12913-018-3277-5 29914477 PMC6006704

[42] Adegboye MB, Zakari S, Ahmed BA, Olufemi GH, Knowledge OGH Knowledge, awareness and practice of infection control by health care workers in the intensive care units of a tertiary hospital in Nigeria. Afr Health Sci 2018; 18: 72–8. doi: 10.4314/ahs.v18i1.11 29977260 PMC6016975

[43] Stein AD, Makarawo TP, Ahmad MFR A survey of doctors' and nurses' knowledge, attitudes and compliance with infection control guidelines in Birmingham teaching hospitals. J Hosp Infect 2003; 54: 68–73. doi: 10.1016/S0195-6701(03)00074-4 12767850

[44] The British Institute of Radiology. British Institute of Radiology online survey of imaging and oncology professionals. 2020 Available from: https://www.bir.org.uk/media/425533/covid-19_-_survey_-_imaging___oncology_professionals_may_2020.pdf.

[45] Shevlin M, McBride O, Murphy J, Miller JG, Hartman TK, Levita L, et al Anxiety, depression, traumatic stress, and COVID-19 related anxiety in the UK general population during the COVID-19 pandemic. PsyArXiv 2020;. 10.1192/bjo.2020.109 PMC7573460 33070797

[46] Blake H, Bermingham F Psychological well-being for healthcare workers: mitigating the impact of covid-19. The University of Nottingham. Version 1.0. 2020 Available from: https://www.nottingham.ac.uk/toolkits/play_22794 [2020 April 25].

[47] The Society of Radiographers. Well-being, emotional and mental health support and resources. 2020 Available from: https://covid19.sor.org/wellbeing,-emotional-and-mental-health/support-and-resources/ [2020 April 20].

[48] Lewis EF, Hardy M, Snaith B An analysis of survey reporting in the imaging professions: is the issue of non-response bias being adequately addressed? Radiography 2013; 19: 240–5. doi: 10.1016/j.radi.2013.02.003

[49] Baruch Y, Holtom BC Survey response rate levels and trends in organizational research. Hum Relat 2008; 61: 1139–60. doi: 10.1177/0018726708094863

[50] NHS. Gender in the NHS infographic. 2020 Available from: https://www.nhsemployers.org/case-studies-and-resources/2019/05/gender-in-the-nhs-infographic [2020 April 20].

